# The aberrant expression in epithelial cells of the mesenchymal isoform of FGFR2 controls the negative crosstalk between EMT and autophagy

**DOI:** 10.1111/jcmm.16309

**Published:** 2021-02-20

**Authors:** Danilo Ranieri, Monica Nanni, Luisa Guttieri, Maria Rosaria Torrisi, Francesca Belleudi

**Affiliations:** ^1^ Department of Clinical and Molecular Medicine Sapienza University of Rome Rome Italy; ^2^ Tissue Biology Research Unit Department of Surgery University Children's Hospital Zurich Switzerland; ^3^ S. Andrea University Hospital Rome Italy

**Keywords:** autophagy, EMT, FGFR2c, FGFRs, signalling, tumorigenesis

## Abstract

Signalling of the epithelial splicing variant of fibroblast growth factor receptor 2 (FGFR2b) triggers both differentiation and autophagy, while the aberrant expression of the mesenchymal FGFR2c isoform in epithelial cells induces impaired differentiation, inhibition of autophagy as well as the induction of the epithelial‐mesenchymal transition (EMT). In light of the widely proposed negative loop linking autophagy and EMT in the early steps of carcinogenesis, here we investigated the possible involvement of FGFR2c aberrant expression and signalling in orchestrating this crosstalk in human keratinocytes. Biochemical, molecular, quantitative immunofluorescence analysis and in vitro invasion assays, coupled to the use of specific substrate inhibitors and transient or stable silencing approaches, showed that AKT/MTOR and PKCε are the two hub signalling pathways, downstream FGFR2c, intersecting with each other in the control of both the inhibition of autophagy and the induction of EMT and invasive behaviour. These results indicate that the expression of FGFR2c, possibly resulting from FGFR2 isoform switch, could represent a key upstream event responsible for the establishment of a negative interplay between autophagy and EMT, which contributes to the assessment of a pathological oncogenic profile in epithelial cells.

## 1 INTRODUCTION

The fibroblast growth factor receptors (FGFR1‐4) are receptor tyrosine kinases regulating key processes, such as cell proliferation, differentiation, migration and survival.^1^ The epithelial isoform of fibroblast growth factor receptor 2 (FGFR2b) isoform controls the entire program of keratinocytes differentiation,^2^, ^3^ while the FGFR2 isoform switch and the consequent aberrant expression of the mesenchymal FGFR2c isoform in epithelial context induces the impairment of differentiation, EMT and tumorigenic features,^4^, ^5^ mainly involving PKCε signalling.^6^

Context‐dependent different roles in cancer have been recently also proposed for the degradative pathway of autophagy.^7^ In fact, while during metastatic spreading autophagy appears to sustain EMT, during the early steps of tumorigenesis the two processes appear to be linked to a negative loop.^8^ Concerning autophagy, we previously highlighted that, while FGFR2b signalling enhances the physiological, positive interplay between this process and keratinocyte differentiation via the activation of JNK1 signalling,^9^, ^10^ FGFR2 isoform switch and the consequent aberrant expression and signalling of FGFR2c inhibit the autophagic process, via the activation of the canonical AKT/MTOR pathway.^11^

On the light of all these evidences, we wondered if FGFR2c aberrant expression might lead to the acquisition of tumorigenic features not only by activating a complex oncogenic signalling network engaging several players, including PKCε, but also by upstream establishing and controlling a negative crosstalk between EMT and autophagy.

## 2 MATERIAL AND METHODS

### 2.1 Cells and treatments

The human keratinocyte cell line HaCaT, stably expressing FGFR2c (pBp‐FGFR2c), or the empty vector (pBp) was cultured in DMEM 10% FBS plus antibiotics. For MTOR and PKCε silencing, clones were transiently transfected with MTOR small interfering RNA (MTOR siRNA) (Santa Cruz Biotechnology, Inc, Santa Cruz, CA, USA; SC35409), or stably transfected with PKCε Plasmide shRNA (h) vector (Santa Cruz; SC‐36251‐SH), or an unrelated siRNA/shRNA as a control, using Lipofectamine 2000 transfection reagent (Life Technologies, Carlsbad, CA, USA; 11668‐019). For growth factor stimulation, cells were left untreated or incubated with FGF2 (PeproTech, London, BFGF 100‐188) 100 ng/mL for 24 hours at 37°C. For inhibition of FGFR2c tyrosine kinase activity, cells were pre‐incubated with a specific FGFR2 tyrosine kinase inhibitor, SU5402 25 µmol/L (Calbiochem, Nottingham, UK; 572 630) for 1 hour before treatments with growth factors (GFs). To inhibit AKT or MTOR, cells were incubated with AKT‐specific inhibitor AKT‐I‐1/2 (1 µmol/L; Calbiochem, 124 005) or with the specific MTOR inhibitor rapamycin (100 nmol/L; Cell Signaling Technology, Beverly, MA, USA; 9904), respectively, for 1 hour at 37°C before being treated with FGF2 in the presence of each inhibitor.

### 2.2 Invasion assay

Migration assay was performed using 24‐well transwell migration Boyden chambers (8 μm pore size; Costar, Cambridge, MA, USA) pre‐coated with matrigel (dilution 1:2 in DMEM; BD Biosciences, Bedford, MA, USA) as reported.^4^ Quantitative analysis was assessed counting for each sample the migrated cells in 10 microscopic fields (objective used: 20X) from three independent experiments. Results have been expressed as mean values ± SD. p values were calculated using Student's *t* test, and significance level has been defined as *P* > .05.

### 2.3 Immunofluorescence

HaCaT clones, grown on coverslips, were processed as previously reported.^4^ The following antibodies were used: mouse monoclonal anti‐LC3 (1:100 in PBS, 5F10 Nanotools, Teningen, Germany, 0231); goat anti‐mouse IgG‐Alexa Fluor 488 (1:200 in PBS, Life Technologies, Carlsbad, CA, A11001). Nuclei were stained with DAPI (Sigma‐Aldrich, D9542). Fluorescence signals were analysed as previously reported.^4^ Results are shown as means ± standard error (SE). Student's *t* test was performed, and significance levels have been defined as *P* < .05.

### 2.4 Western blot analysis

Cells were lysed, and total proteins were collected, separated and blotted as reported.^4^ The membranes were incubated with anti‐SQSTM1 (BD Bioscience, San Josè, CA, USA, 610 833), anti‐p‐FGFR (55H2, Y653/654, Cell Signaling, 3476S), anti‐p‐MTOR (Ser 2448, Cell Signaling, 5536S), anti‐E‐cadherin (NCH‐38, Dako, Carpinteria, CA, USA), anti‐β4‐integrin (7, Santa Cruz Biotechnology, sc‐135,950), anti‐N‐cadherin (Sigma‐Aldrich, Saint Louis, MO, USA, C3865) monoclonal antibodies or with anti‐LC3 (MBL, Woburn, MA, PD014), p‐PKCε (Ser729, Abcam, Cambridge, UK, ab63387), anti‐p‐AKT (Ser 473; Cell Signaling, 9271), anti‐p‐S6K (ser 371, Cell Signaling, #9208), polyclonal antibodies, followed by enhanced chemiluminescence (ECL) detection (Thermo Scientific, Rockford, IL, USA; 34 580). The membranes were rehydrated by washing in PBS/Tween‐20, stripped with 100 mmol/L β‐mercaptoethanol and 2% SDS for 30 minutes at 55°C and probed again with, anti‐Bek (C17, Santa Cruz Biotechnology, sc‐122), anti‐PKCε (Abcam, ab124806), anti‐AKT (H‐136; Santa Cruz Biotechnology, sc‐8312), anti‐ α/β‐Tubulin (Cell Signaling, 2148S), anti‐S6K (Cell Signaling, #9202) polyclonal antibodies or with anti‐MTOR (7C10, Cell Signaling, 2983S), anti‐ACTB (Sigma‐Aldrich, A5441), anti‐GAPDH (6C5, Santa Cruz Biotechnology, sc‐32233) monoclonal antibody to estimate the protein equal loading. Densitometric analysis was performed using Quantity One Program version 4.6.8 (Bio‐Rad). The resulting values from three different experiments were normalized, expressed as fold increase respect to the control value and reported in graph as mean values ± standard deviation (SD). Student's *t* test was performed, and significance levels have been defined as *P* < .05.

### 2.5 Primers

Oligonucleotide primers necessary for target genes and the housekeeping gene were chosen by using the online tool Primer‐BLAST ^4^ and purchased from Invitrogen (Invitrogen, Carlsbad, CA, USA). The following primers were used: for the Snail1 target gene: 5′‐GCTGCAGGACTCTAATCCAGA‐3′ (sense), 5′‐ATCTCCGGAGGTGGGATG‐3′ (antisense); for the STAT3 target gene: 5′‐CAGAGATGTGGGAATGGGGG‐3′ (sense), 5′‐ TGGCAAGGAGTGGGTCTCTA‐3′ (antisense); for the FRA1 target gene: 5′‐GCAGGCGGAGACTGACAAA‐3′ (sense), 5′‐ GATGGGTCGGTGGGCTTC‐3′ (antisense); and for the 18S rRNA housekeeping gene: 5’‐CGAGCCGCCTGGATACC‐3’ (sense) and 5’‐CATGGCCTCAGTTCCGAAAA‐3’ (antisense).

### 2.6 RNA extraction and cDNA synthesis

RNA was extracted and retrotranscribed using the iScriptTM cDNA synthesis kit (Bio‐Rad, 170‐8891) as previously reported.^4^

### 2.7 PCR amplification and real‐time quantitation

Real‐time RT‐PCR was performed using the iCycler real‐time detection system (iQ5 Bio‐Rad) with optimized PCR conditions as reported.^4^ Results are reported as mean values ± SE from three different experiments in triplicate. Student's *t* test was performed, with significance levels defined as p values < 0.05. *

## 3 RESULTS AND DISCUSSION

### 3.1 The forced reactivation of autophagy reverses FGFR2c‐induced EMT program and inhibits receptor‐mediated cell invasion

**FIGURE 1:**
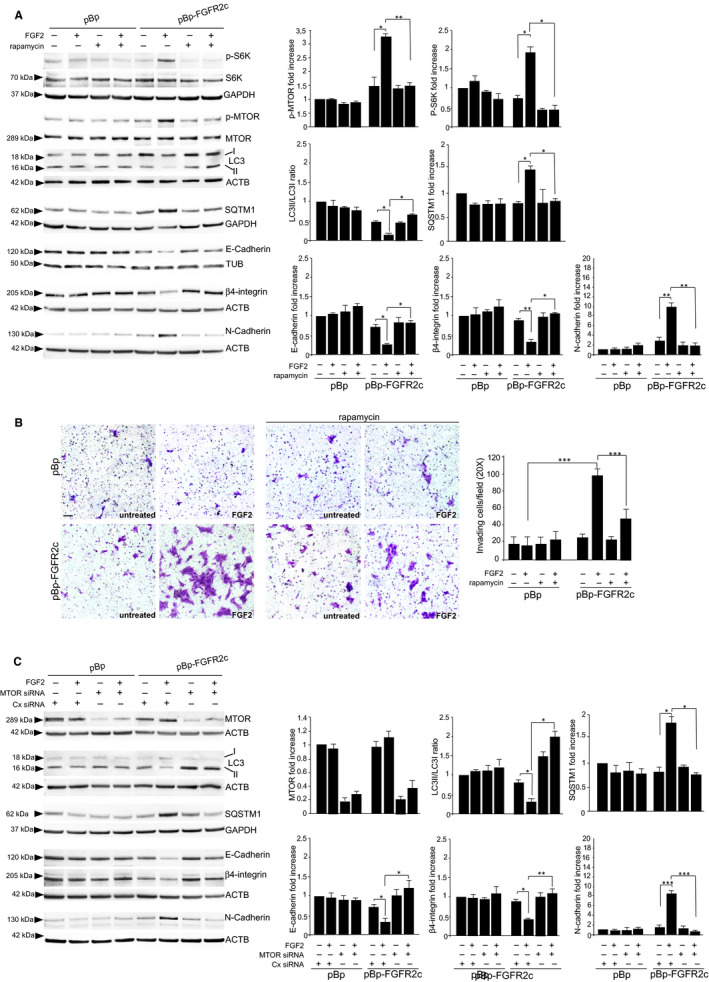
Reactivation of MTOR‐dependent autophagy negatively impact on FGFR2c‐mediated EMT and cell invasion. A, HaCaT pBp‐FGFR2c and HaCaT pBp clones were left untreated or stimulated with FGF2 in presence or not of rapamycin. Western blot analysis shows that, only in FGF2‐stimulated pBp‐FGFR2c clones, rapamycin negatively interferes with MTOR and S6K phosphorylation, with LC3‐II decrease (turning it into an increase) and SQSTM1 accumulation and reverses the repression of the epithelial markers E‐cadherin and β4‐integrin, as well as the appearance of the mesenchymal marker N‐cadherin. For the densitometric analysis, the values from 3 independent experiments were normalized, expressed as fold increase and reported in graph as mean values ± standard deviation (SD). Student *t* test was performed as reported in Materials and Methods, and significance levels have been defined as *P* < .05: *P < .05, ** *P* < .01. B, HaCaT clones were seeded on matrigel pre‐coated transwell Boyden chamber filters and FGF2 was added in the bottom chamber, in the presence or not of rapamycin, to stimulate cell chemotaxis. Results shows that, in FGFR2c cultures, the increase of the number of invading cells induced by FGF2 is counteracted by rapamycin. Quantitative analysis was assessed as reported in Materials and Methods. Results are expressed as mean values ± standard deviation (SD). Student's *t* test was performed as reported in Materials and Methods, and significance level has been defined as *P* < .05: ***P < .001. Bar: 50 μm. C, HaCaT clones were transiently transfected with MTOR siRNA or with an unrelated siRNA (Cx RNA), as negative control, and then left untreated or stimulated with FGF2 as above. Western blot analysis shows that MTOR silencing reverses the decrease of LC3‐II, the accumulation of SQSTM1 and the modulation of the epithelial markers E‐cadherin and β4‐integrin and that of the mesenchymal marker N‐cadherin induced by FGF2 stimulation in FGFR2c expressing clones. Densitometric analysis was performed as above. *P < .05, ** *P* < .01

Since MTOR is the main pathway involved in the inhibition of autophagy induced by aberrant FGFR2c expression in human keratinocytes,^11^ we first assessed if a negative crosstalk between receptor‐controlled autophagy and EMT does exist in these cells using rapamycin, the widely accepted general inhibitor of MTOR‐dependent autophagy. Western blot analysis, performed in HaCaT pBp‐FGFR2c clones and pBp controls,^4^ left untreated or stimulated with FGF2, the ligand which does not bind to FGFR2b, but is able to activate other FGFRs including FGFR2c, showed that rapamycin was able to interfere with the phosphorylation of MTOR at Ser 2448, as well as with that of its downstream substrate S6K, at Ser 371, both induced only in pBp‐FGFR2c clones by ligand stimulation (Figure 1A). In these cells, rapamycin also turned the decrease of the widely recognized autophagic marker LC3‐II into an increase (Figure 1A). As previously speculated by us, this effect is possibly attributable to the negative interplay between MTOR pathway and JNK1 signalling that causes JNK1‐dependent activation of autophagy in consequence of MTOR signalling shut‐off.^11^ In addition to the impact on LC3‐II decrease, rapamycin also reversed the accumulation of the autophagic substrate SQSTM1/p62, detectable only in FGFR2c clones stimulated by FGF2, confirming the reactivation of the autophagic flux (Figure 1A), Then, we focused our attention on EMT markers expression, observing that rapamycin efficiently reversed the decrease of the epithelial markers E‐cadherin and β4‐integrin, as well as the appearance of the mesenchymal marker N‐cadherin, both caused in pBp‐FGFR2c clones by FGF2 treatment (Figure 1A).

We then investigated the impact of MTOR inhibition on the invasion ability, displayed by pBp‐FGFR2c clones,^4^, ^5^ using the in vitro assay of matrigel pre‐coated transwell Boyden chambers. Upon cell seeding, FGF2 was added in the bottom chamber, in the presence or not of rapamycin, to stimulate cell chemotaxis. The results showed that the significant increase of invading cells, observed in FGFR2c cultures only in response to FGF2 (Figure 1B), was clearly impaired by the presence of rapamycin (Figure 1B). These results are consistent with the recent findings by Bell and coworkers, highlighting how the inhibition of autophagy, which impairs Met receptor traffic, is required for HGF‐dependent invasive behaviour in several tumour contexts.^12^

In order to further assess the outcome of forced reactivation of MTOR‐dependent autophagy on FGFR2c‐mediated EMT in epithelial context, MTOR protein depletion was carried out in HaCaT clones by specific siRNA transfection. Western blot analysis showed that, similarly to what observed in the presence of rapamycin, in HaCaT pBp‐FGFR2c cells stimulated with FGF2, MTOR silencing was not only sufficient to reverse the decrease of LC3‐II in an increase and to block SQSTM1 accumulation (Figure 1C), confirming the reactivation of autophagy, but also was effective in counteracting the FGF2‐mediated modulation of the epithelial/mesenchymal markers (Figure 1C), confirming the impairment of EMT program.

Since AKT is the substrate acting upstream MTOR in FGFR2c‐mediated inhibition of autophagy,^11^ we also checked the effects of its inhibition on FGFR2‐driven EMT. Western blot analysis showed that AKT signalling shut‐off by AKT‐I‐1/2 inhibitor^11^ efficiently reversed the decrease of LC3‐II levels, the accumulation of SQSTM1 and the modulation of the EMT‐related markers induced by FGF2 only in FGFR2c expressing clones (Figure S1). These results confirm the involvement of the entire AKT/MTOR signalling pathway at the crossroad between autophagy regulation and EMT processes.

Since FGFR2c‐triggered EMT is driven by PKCε‐dependent induction of Snail1, STAT3 and FRA1,^4^, ^6^ we wondered if and how the forced reactivation of autophagy could impact on the expression of these EMT‐related transcription factors. Real‐time RT‐PCR showed that both MTOR silencing via siRNA (Figure S2A) and PKCε stable depletion by shRNA (Figure S2B) were able to counteract the increase of mRNA levels of all these transcription factors, evident in pBp‐FGFR2c clones in response to FGF2 (Figure S2 A, B). Thus, the forced reactivation of the autophagic process appears to negatively affect the induction of EMT‐related transcription factors in a comparable way to PKCε signalling shut‐off.

### 3.2 Selective PKCε shut‐off efficiently reverses the negative impact of FGFR2c signalling on autophagy

**FIGURE 2:**
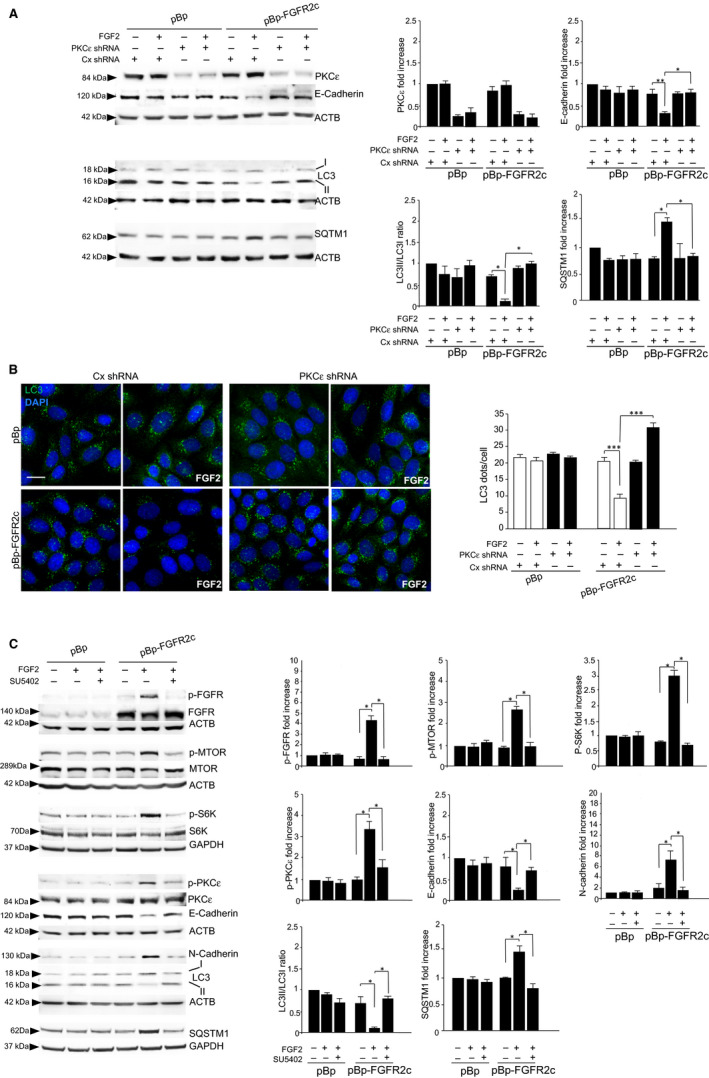
PKCε signalling shut‐off restores the autophagic process and FGFR2c signalling underly EMT/autophagy negative crosstalk. HaCaT pBp‐FGFR2c and HaCaT pBp clones were stably transfected with PKCε shRNA or with an unrelated shRNA, as negative control, and then left untreated or stimulated with FGF2 as above. A, Western blot analysis shows that PKCε depletion reverses all the effects induced by FGF2 on the expression of E‐cadherin, LC3‐II and SQSTM1 in HaCaT pBp‐FGFR2c clones. Densitometric analysis and Student *t* test were performed as reported in Figure 1A. *P < .05, **P < .01. B, Quantitative immunofluorescence analysis shows that PKCε depletion reverses the decrease of LC3 positive dots per cell induced in HaCaT pBp‐FGFR2c cells by the stimulated with FGF2. Quantitative analysis of LC3 positive dots per cell was performed as described in Materials and Methods, and the results are expressed as mean values ± standard errors (SE). Student's *t* test was performed, and significance level was defined as *P* < .05: *** *P* < .001. Bar: 20 µm. C, HaCaT pBp‐FGFR2c and HaCaT pBp clones were left untreated or stimulated with FGF2 in presence or not of the FGFR2 kinase inhibitor SU5402. Western blot analysis shows that SU5402 abolishes FGFR2c, PKCε, MTOR and S6K phosphorylation, and reverses both the modulation of E‐cadherin and N‐cadherin markers, the repression of LC3‐II and the accumulation of SQSTM1 induced by FGF2 in HaCaT pBp‐FGFR2c clones. Densitometric analysis and Student *t* test were performed as above. *P < .05, ** *P* < .01

The possibility that FGFR2c could orchestrate a fine interplay between autophagy and EMT in epithelial context is also sustained by the evidence that protein kinase C isozymes, including PKCε, are also key regulators of the autophagic pathway.^13^ Thus, we investigated the possible contribution of PKCε signalling on FGFR2c‐mediated repression of the autophagic process in human keratinocytes by shRNA approaches. The impairment of FGF2‐induced EMT program in FGF2‐stimulated FGFR2c clones after PKCε depletion was confirmed by the recovery of the epithelial marker E‐cadherin (Figure 2A), while LC3‐II increase indicated the activation of autophagy (Figure 2A). In addition, the expected accumulation of the autophagy substrate SQSTM1 in FGFR2c clones stimulated by FGF2 was significantly dampened by PKCε depletion (Figure 2A), suggesting a reactivation of the autophagic flux. Finally, quantitative immunofluorescence approaches showed that the expected reduction of LC3 positive dots per cell in HaCaT pBp‐FGFR2c clones stimulated with FGF2^11^ (Figure 2B) was completely reversed by stable depletion of PKCε, resulting in a visible increase (Figure 2B). Thus, PKCε signalling appears to be involved in the inhibition of autophagy orchestrated by FGFR2c, when this receptor is aberrantly expressed in human keratinocytes. Our results are consistent with previous data showing that PKCε is involved in the suppression of the autophagic process in glioblastoma cells.^14^ In addition, the transcription factor STAT3, activated downstream PKCε during FGFR2c‐induced EMT,^6^ has been found involved not only in the triggering of EMT program,^6^, ^15^ but also in the inhibition of autophagy.^16^ Despite these supporting studies, our current results seem apparently in contrast with the recent findings reported by Basu, which indicate a key role of PKCε in promoting autophagic process in metastatic breast cancer cells.^17^ This discrepancy would be explained considering the hypothesis that FGFR2c plays its oncogenic role in the early steps of tumour development,^4^, ^5^, ^6^ further confirming the dual and opposite contribution of autophagy in different (early and advanced‐metastatic) steps of carcinogenesis.^8^ In fact, it has been proposed that autophagy can play a ‘double‐edged sword’ role on EMT, repressing the process in early, but inducing it in late, stages of tumorigenesis.^8^

As last aim, to confirm the central role of FGFR2c in regulating all the observed, intersected effects between autophagy and EMT, we used the FGFR2 tyrosine kinase inhibitor SU5402. In HaCaT pBp‐FGFR2c clones, the presence of SU5402 was sufficient to abolish all the responses to FGF2, not only in terms of FGFR2c, PKCε and MTOR/S6K phosphorylation (Figure 2C), as expected,^6^, ^11^ but also in term of E‐cadherin/N‐cadherin modulation and repression of LC3 ‐II, as well as SQSTM1 accumulation (Figure 2C). These findings appear to confirm the upstream role of FGFR2c in the regulation of EMT/autophagy crosstalk.

Overall, our results represent the first indication that, at least in the context of human keratinocytes, the aberrant expression of FGFR2c, usually stemming from altered FGFR2 isoform switch, could be the upstream event leading to the activation of oncogenic signalling pathways intersecting with each other and cooperating in the establishment of the negative loop between EMT and autophagy, which contributes to the early steps of tumour development.

## CONFLICT OF INTEREST

The authors declare that they have no competing interests.

## AUTHOR CONTRIBUTION

**Danilo Ranieri:** Conceptualization (equal); Investigation (lead); Writing‐original draft (lead). **Monica Nanni:** Data curation (equal); Investigation (equal). **Luisa Guttieri:** Investigation (equal). **Maria Rosaria Torrisi:** Conceptualization (equal); Writing‐review & editing (equal). **Francesca Belleudi:** Conceptualization (equal); Project administration (lead); Writing‐review & editing (lead).

## Supporting information

Fig S1

Fig S2

## References

[1] Latko M , Czyrek A , Porębska N , Kucińska M , Otlewski J , Zakrzewska, M , Opaliński, Ł . Cross‐talk between fibroblast growth factor receptors and other cell surface proteins. Cells. 2019;8(5):455. 10.3390/cells8050455 PMC6562592 31091809

[2] Purpura V , Belleudi F , Caputo S , Torrisi MR . HPV16 E5 and KGFR/ FGFR2b interplay in differentiating epithelial cells. Oncotarget. 2013;4:192‐205. 23545251 10.18632/oncotarget.803 PMC3712566

[3] Rosato B , Ranieri D , Nanni M , Torrisi MR , Belleudi F . Role of FGFR2b expression and signaling in keratinocyte differentiation: Sequential involvement of PKCδ and PKCα. Cell Death Dis. 2018;9:565. 29752438 10.1038/s41419-018-0509-x PMC5948219

[4] Ranieri D , Rosato B , Nanni M , Magenta A , Belleudi F , Torrisi MR . Expression of the FGFR2 mesenchymal splicing variant in epithelial cells drives epithelial‐mesenchymal transition. Oncotarget. 2016;7:5440‐5460. 26713601 10.18632/oncotarget.6706 PMC4868697

[5] Ranieri D , Rosato B , Nanni M , Belleudi F , Torrisi MR . Expression of the FGFR2c mesenchymal splicing variant in human keratinocytes inhibits differentiation and promotes invasion. Mol. Carcinog. 2018;57:272‐283. 29068468 10.1002/mc.22754 PMC5813158

[6] Ranieri D , Nanni M , Persechino F , Torrisi MR , Belleudi F . Role of PKCε in the epithelial‐mesenchymal transition induced by FGFR2 isoform switch. Cell Commun Signal. 2020;18:76. 32429937 10.1186/s12964-020-00582-1 PMC7238605

[7] Kimmelman AC , White E . Autophagy and tumor metabolism. Cell Metab. 2017;25:1037‐1043. 28467923 10.1016/j.cmet.2017.04.004 PMC5604466

[8] Chen HT , Liu H , Mao MJ , et al. Crosstalk between autophagy and epithelial‐mesenchymal transition and its application in cancer therapy. Mol Cancer. 2019;18:101. 31126310 10.1186/s12943-019-1030-2 PMC6533683

[9] Nanni M , Ranieri D , Raffa S , Torrisi MR , Belleudi F . Interplay between FGFR2b‐induced autophagy and phagocytosis: Role of PLCγ‐mediated signaling. J. Cell Mol. Med. 2018;22:668‐683. 28994193 10.1111/jcmm.13352 PMC6193413

[10] Nanni M , Ranieri D , Rosato B , Torrisi MR , Belleudi F . Role of fibroblast growth factor receptor 2b in the cross talk between autophagy and differentiation: Involvement of Jun N‐terminal protein kinase signaling. Mol Cell Biol. 2018;38:e00119‐e218. 29685904 10.1128/MCB.00119-18 PMC6002692

[11] Nanni M , Ranieri D , Persechino F , Torrisi MR , Belleudi F . The aberrant expression of the mesenchymal variant of FGFR2 in the epithelial context inhibits autophagy. Cells. 2019;8:653. 10.3390/cells8070653 PMC6678203 31261937

[12] Bell ES , Pinto Coelho P , Ratcliffe CDH . LC3C‐mediated autophagy selectively regulates the Met RTK and HGF‐stimulated migration and invasion. Cell Rep. 2019;29:4053‐4068. 31851933 10.1016/j.celrep.2019.11.063

[13] Kaleli HN , Ozer E , Kaya VO , Kutlu O . Protein kinase C isozymes and autophagy during neurodegenerative disease progression. Cells. 2020;9:553. 10.3390/cells9030553 PMC7140419 32120776

[14] Toton E , Romaniuk A , Konieczna N , Hofmann J , Barciszewski J , Rybczynska M . Impact of PKCε downregulation on autophagy in glioblastoma cells. BMC Cancer. 2018;18:185. 29439667 10.1186/s12885-018-4095-1 PMC5811983

[15] Wendt MK , Balanis N , Carlin CR , Schiemann WP . STAT3 and epithelial‐mesenchymal transitions in carcinomas. JAKSTAT. 2014;3:e28975. 24843831 10.4161/jkst.28975 PMC4024059

[16] You L , Wang Z , Li H , et al. The role of STAT3 in autophagy. Autophagy. 2015;11:729‐739. 25951043 10.1080/15548627.2015.1017192 PMC4509450

[17] Basu A . Regulation of autophagy by Protein Kinase C‐ε in breast cancer cells. Int J Mol Sci. 2020;21. 10.3390/ijms21124247 PMC7352677 32549199

