# Supplementary material for: The aberrant expression in epithelial cells of the mesenchymal isoform of FGFR2 controls the negative crosstalk between EMT and autophagy
Source: J Cell Mol Med. 2021 Feb 20;25(8):4166–72. doi: 10.1111/jcmm.16309 (PMC8051744; doi:10.1111/jcmm.16309)
Supplement: Supplementary file 1 — Fig S1 [file JCMM-25-4166-s001.pdf]

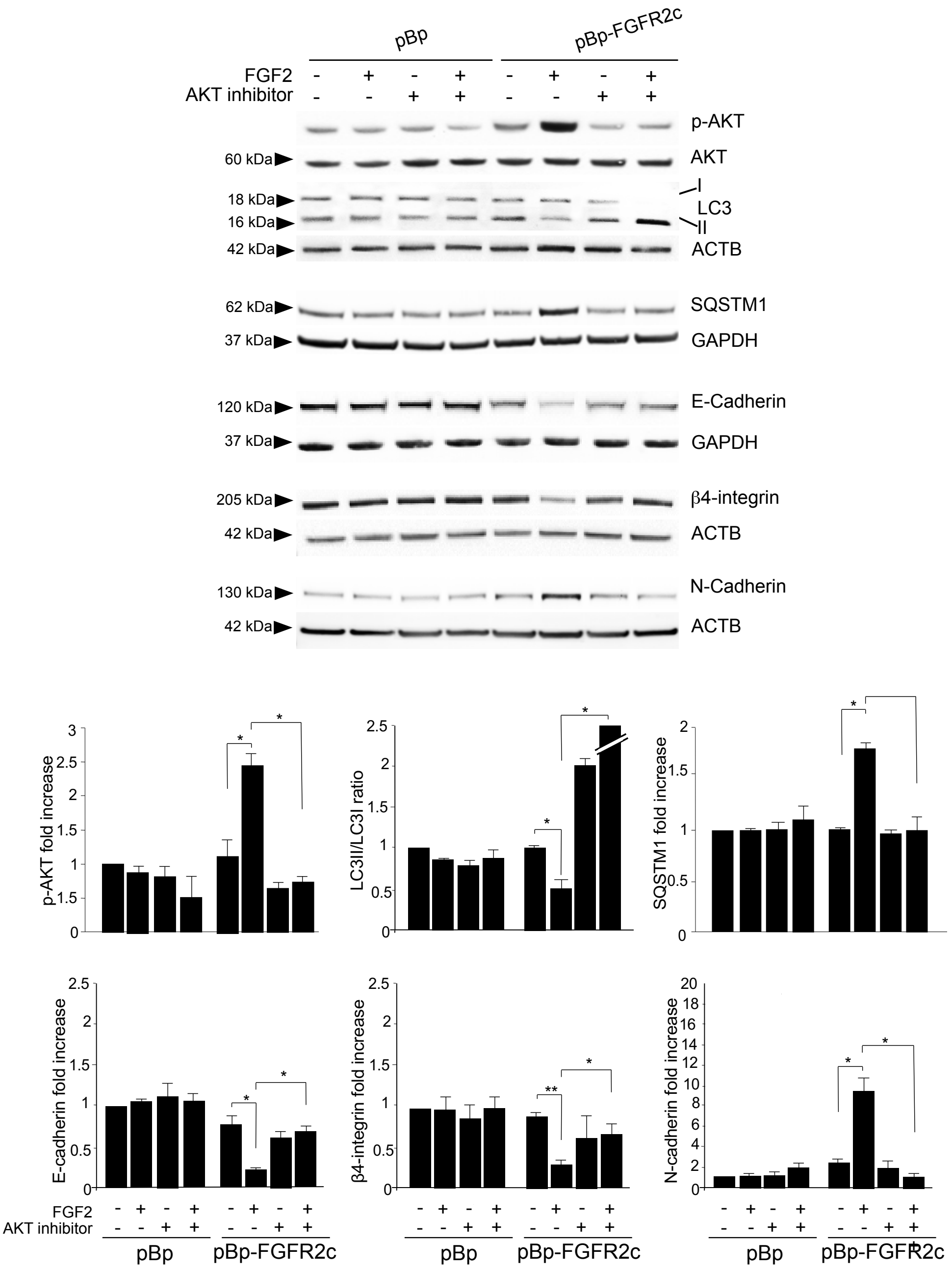

Figure S1

The inhibition of AKT signaling represses FGFR2c-induced EMT. HaCaT pBp-FGFR2c and HaCaT pBp clones were left untreated or stimulated with FGF2 in presence or not of AKT-I-1/2 inhibitor. Western blot analysis shows that AKT inhibitor reverses the decrease of LC3-II levels, the accumultion of SQSTM1 and the modulation of all the EMT-related markers induced by FGF2 stimulation in FGFR2c expressing clones. The densitometric analysis and Student t test were performed as reported in Figure 1a: *p<0.05, ** p<0.01.
