# Supplementary material for: The aberrant expression in epithelial cells of the mesenchymal isoform of FGFR2 controls the negative crosstalk between EMT and autophagy
Source: J Cell Mol Med. 2021 Feb 20;25(8):4166–72. doi: 10.1111/jcmm.16309 (PMC8051744; doi:10.1111/jcmm.16309)
Supplement: Supplementary file 2 — Fig S2 [file JCMM-25-4166-s002.pdf]

A

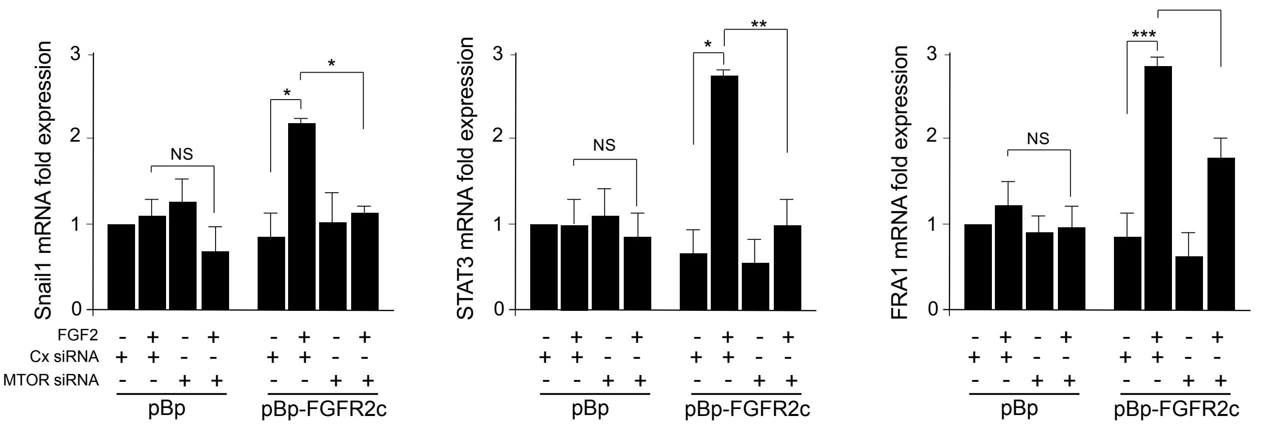

B

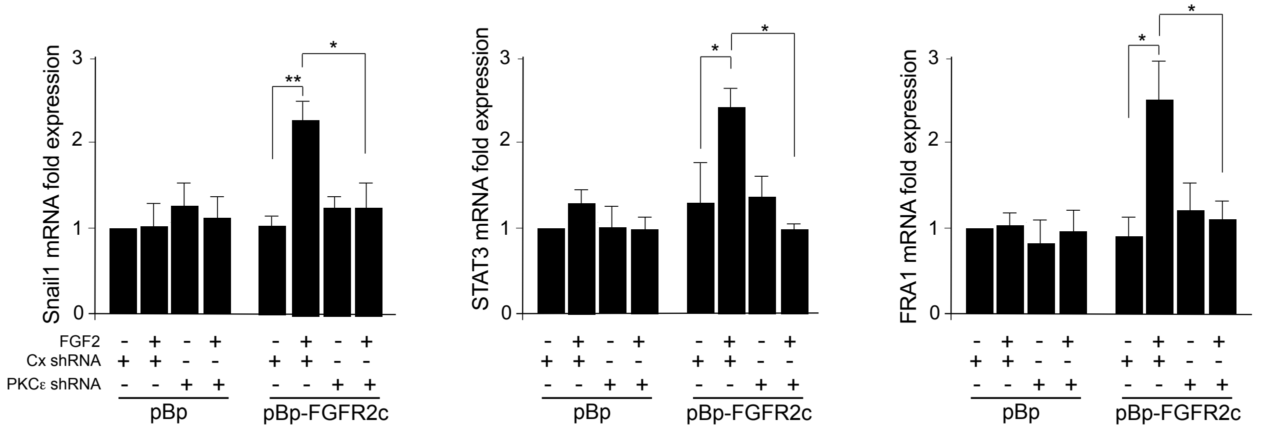

Figure S2

MTOR silencing and PKCε depletion similarly interfere with FGFR2c-mediated induction of the EMT-related transcription factors Snail1, STAT3 and FRA1. HaCaT pBp-FGFR2c and HaCaT pBp clones were transiently transfected with MTOR siRNA (A) or stably transfected with PKCε shRNA (B) and then left untreated or stimulated with FGF2 as above. Unrelated siRNA (Cx siRNA) and shRNA (Cx shRNA) were respectively used as negative controls. Both MTOR transient silencing (A) and PKCε stable depletion (B) counteract the increase of Snail1, STAT3 and FRA1 mRNA levels, induced in FGFR2c expressing clones in response to FGF2 stimulation. Results are expressed as mean value ± SD. Student's t test was performed as reported in Materials and Methods and significance level was defined as $p<0.05$: *$p<0.05$, ** $p<0.01$.
